# Perceptions and Attitudes Toward Life-Sustaining Treatment Communication: A Comparison Between Physicians and Surrogates

**DOI:** 10.3390/healthcare13212707

**Published:** 2025-10-27

**Authors:** Yang Liang, Zhen Ren, Aixiang Song, Shu Li

**Affiliations:** 1 Department of Emergency Medicine, Peking University Third Hospital, No. 49 North Garden Road, Haidian District, Beijing 100191, China; 2 Key Laboratory of Molecular Cardiovascular Sciences, Ministry of Education, No. 49 North Garden Road, Beijing 100191, China

**Keywords:** life-sustaining treatment, shared decision-making, doctor-patient communication

## Abstract

**Background:** Effective shared decision-making (SDM) for life-sustaining treatment (LST) requires alignment between physicians and surrogates. However, discrepancies in perceptions and communication may hinder ethically sound decisions. This study aimed to compare the perceptions and attitudes of physicians and surrogates toward SDM for LST in a Chinese hospital setting. **Methods:** This pre-planned secondary analysis included data from two cross-sectional surveys administered to physicians and surrogates. Participants were 325 surrogates of critically ill adult patients admitted to the Emergency Intensive Care Unit (EICU) of a tertiary teaching hospital and 351 physicians from hospitals in Beijing. Survey items assessed triggers and preferred models of decision-making, disclosure practices, perceived decisional capacity, and factors influencing LST decisions. Statistical comparisons were performed using appropriate tests for categorical data. **Results:** Although a majority in both groups nominally preferred SDM (physicians: 52.7%; surrogates: 44.3%; *p* = 0.155), significant discrepancies emerged across other domains. Physicians were more likely than surrogates to initiate LST discussions earlier (88.0% vs. 75.3%; *p* < 0.001). Perceived understanding differed markedly: 87.7% of surrogates rated their comprehension as “good” or “excellent”, whereas 73.8% of physicians rated surrogate understanding as “fair” or “poor” (*p* < 0.001). Surrogates expressed a stronger preference for receiving quantitative prognostic information and decision-support tools. Most physicians (94.9%) reported directing consent discussions primarily to families, with limited patient involvement. Priorities for LST decisions diverged: physicians emphasized clinical indicators such as prognosis (96.0%) and comorbidities (91.7%), whereas surrogates emphasized patient age (72.0%). **Conclusions:** Marked discordances exist between physicians and surrogates in their perceptions and practices regarding SDM for LST in China. Differences in communication strategies, informational expectations, and decision-making priorities underscore the need for contextually adapted interventions, such as structured communication tools and culturally sensitive clinician training, to bridge these gaps and support ethically aligned decision-making.

## 1. Introduction

Shared decision-making (SDM) is increasingly recognized as a cornerstone of ethical clinical practice, particularly in critical care settings where decisions are high-stakes, emotionally charged, and often time-sensitive. It is recommended as “best practice” to achieve patient value-congruent and well-informed decisions, and to reduce decisional conflict, decision regret, and potentially surrogate psychological distress [1]. The 2003 Challenges in End-of-Life Care in the ICU consensus statement, generated from expert opinion, strongly advocates a shared decision-making model in which surrogate decision-makers and physicians share the responsibility of making decisions on behalf of incapacitated ICU patients [2].

However, the successful implementation of SDM is fraught with significant barriers. Effective communication and decision-making require not only a complex integration of relevant conceptual knowledge of ethical implications and legal considerations but also considerable communication skills that address the highly charged emotional issues under discussion from medical professionals [3]. Some physicians may be hesitant to accurately characterize prognosis, especially in the context of life-threatening illness [4]. On the other hand, many individuals prefer to leave medical decisions to their provider or have difficulty participating in medical decisions because of cognitive impairment or low health literacy [4]. For patients who lack capacity to make decisions, surrogate decision-makers may not be present or may struggle to accurately interpret patients’ previously expressed wishes [5]. Surrogates may even experience conflict with physicians or have difficulty identifying patients’ treatment preferences [6]. Different surrogate perspectives may provide vastly different information that is potentially critical to patient management and treatment, particularly in vulnerable populations such as the critically ill and cognitively impaired [7]. Current research has theorized about barriers but lacks empirical evidence on how these stakeholders specifically perceive the communication process, their respective roles, and the challenges in achieving collaborative decisions for critically ill patients.

While these challenges are universal, information disclosure, the perception of patient autonomy, and the very nature of collaboration are deeply mediated by cultural context. These cultural values influence how individuals engage with the healthcare system and provide a framework of ethical priorities when making decisions about diagnosis and treatment. Studies indicate that Western societies often prioritize self-determination, individual rights, and full transparency. In contrast, many non-Western cultures, including those in East Asia, are frequently guided by principles of family-determination, a moral responsibility to collective well-being, and a more protective approach to information disclosure [8]. The substitute responsible party is typically defined by a family-centric model of decision-making, where authority derives from kinship hierarchy and informal familial relationships rather than formal legal appointment. China represents a compelling context for studying SDM due to its unique socio-cultural soil, where familial harmony and obligations often take precedence over individual autonomy. In this environment, family members are not merely involved in decision-making; they are frequently the primary recipients of medical information and the central architects of care decisions, acting under the conviction that they are shielding the patient from harm and fulfilling their familial duty [9]. Consequently, although the barriers to SDM are well-documented in Western contexts, it requires critical examination and adaptation to be effective within this collective framework. Despite its importance, there is a scarcity of research directly comparing the specific communication challenges as experienced by physicians and patient surrogates in China regarding the process of serious-illness communication.

Therefore, we performed a pre-planned secondary analysis of previous studies by the same research group. This study aims to describe and analyze the differences in perceptions and attitudes between physicians and the surrogates of critically ill patients in China, testing the hypothesis that significant gaps exist in their understanding of decision-making triggers, information disclosure, and assessments of patient decisional capacity.

## 2. Methods

### 2.1. Study Design and Setting

This was a pre-planned secondary analysis of two previously conducted studies. One study was a single-centre, prospective interventional study aimed at improving the quality of serious-illness conversations regarding life-sustaining treatment (LST). It demonstrated that the use of a structured serious-illness conversation checklist in the Emergency Intensive Care Unit (EICU) at Peking University Third Hospital, a tertiary care academic hospital in Beijing, China, significantly reduced Decisional Conflict Scale (DCS) scores among patients’ surrogates [10,11]. In that study, a cross-sectional survey assessed surrogates’ experiences with the shared decision-making process for LST using a questionnaire that included DCS evaluation. The second study involved a cross-sectional survey of physicians to assess perceived barriers to SDM in the context of LST in China [12]. This secondary analysis focused on comparing the perceptions and attitudes of surrogates and physicians involved in serious-illness communication.

### 2.2. Study Population and Survey Instrument

The previous study of patient surrogates included adult patients (≥18 years) admitted to the EICU and their designated surrogates. Detailed inclusion and exclusion criteria are available in the original publication [11]. In this study, the term ‘surrogate’ or ‘proxy’ refers to the primary informal decision-maker for the incapacitated patient. In accordance with standard clinical practice in China, where formal legal appointment of a healthcare proxy is uncommon, this was typically the patient’s closest available family member (e.g., spouse, adult child, or parent) who was most involved in their care and responsible for communication with the medical team. Eligibility required that the individual self-identified as playing this central decision-making role. The physician cohort consisted of clinicians from the same city who participated in LST-related decision-making and were enrolled in the earlier physician-focused study. Eligible physicians were invited to participate, and only those who provided written informed consent were enrolled in the study. The design of both studies and the development of the survey instruments have been described in detail previously [11,12]. The questionnaire consisted of five domains with 33 questions (3–10 questions in each domain), including demographic variables, overall viewpoints on existing barriers of shared decision-making on life-sustaining treatment, perceived treatment and prognosis, expressed decisional capacity and patient value, and physician communication patterns. In the original instruments, parallel questions were presented to physicians and patient surrogates in Mandarin. The full questionnaire is available as a supplement to the original publication.

Given the differences in topic, setting, and populations between studies, estimating a uniform effect size was not feasible. Although sample sizes in related studies have varied, a minimum sample size of five times the number of survey items is commonly recommended to enhance statistical robustness. Based on consultation with a clinical epidemiologist (ZH), a minimum sample size of 150 observations was defined. Then, we used the EVP (events per variable) method to estimate the sample size. We investigated 12 target factors and calculated a sample size of 240, with the EVP as 20.

### 2.3. Statistical Analysis

Demographic characteristics of all participants were collected via questionnaire. Descriptive statistics were used to summarize participant characteristics. Group comparisons were performed using non-parametric methods. Continuous quantitative variables were analyzed using the Mann–Whitney U test, and categorical variables were assessed using the Chi-square test or Fisher’s exact test, as appropriate. All *p*-values were derived from two-tailed tests, with statistical significance set at *p* < 0.05. Data were analyzed using IBM SPSS Statistics for Windows, version 22.0 (SPSS Science Inc., Chicago, IL, USA).

### 2.4. Ethical Approval

Both original studies were approved by the Peking University Third Hospital Medical Science Research Ethics Committee (approval numbers: IRB-2021-493 and IRB-2021-591). No additional ethical approval was required for this secondary analysis. All participants were informed of the purpose of the survey, and electronic or written informed consent was obtained prior to participation. Participants could proceed with the questionnaire only after providing informed consent.

## 3. Results

### 3.1. Participant Demographic Characteristics

A total of 351 adult patients were admitted to the EICU. Data from 325 surrogates were included in the final analysis; their median age was 49.0 years (interquartile range [IQR], 40–57), and 166 (51.1%) were female. Of the surrogate group, the majority (55.4%, 180/325) held a university degree, representing the largest segment of the population, while individuals with a master’s or doctor’s degree accounted for 12.9% (42/325). The largest occupational group consists of experts, technicians, and related workers, representing 26.8% (87/325) of the sample. Business and service industry workers form another substantial segment at 21.2% (69/325). A total of 351 physicians practicing in the same city were also included. The median age of physicians was 35 years (IQR, 30–40), and 232 (66.1%) were female. The median duration of medical service was 10 years (IQR, 6–16), and 315 (89.7%) worked in tertiary hospitals. The sample comprised 90 senior physicians (25.6%), 115 emergency physicians (32.8%), and 28 ICU physicians (8.0%) (Table 1).

### 3.2. Comparison of Perceptions and Attitudes Between Surrogates and Physicians

#### 3.2.1. Decision-Making Patterns

Shared decision-making was the most commonly preferred approach overall, selected by 52.7% (185/351) of physicians and 44.3% (144/325) of surrogates (*p* = 0.155). Surrogate-led decisions were also prevalent (physicians: 35.9%, 126/351; surrogates: 44.0%, 143/325), while patient-led decisions were rare (physicians: 7.1%, 25/351; surrogates: 7.4%, 24/325). A majority of physicians reported proactively initiating LST discussions during periods of clinical risk, more frequently than surrogates (88.0%, 309/351 vs. 75.3%, 207/275, *p* < 0.001) (Table 2).

#### 3.2.2. Medical Fact Disclosure

Communication content was largely tailored to surrogate requests, cited as a priority by 74.6% (262/351) of physicians and 69.2% (225/325) of surrogates, with no significant difference (*p* = 0.117). Physicians reported offering alternative care plans more frequently than surrogates perceived (93.7%, 329/351 vs. 79.4%, 258/325, *p* < 0.001). While 87.7% (285/325) of surrogates rated their understanding as “good” or “excellent”, 76.4% (268/351) of physicians rated surrogate comprehension as “fair”, “poor”, or “extremely poor” (*p* < 0.001). Surrogates also believed that physicians were more active in clarifying their confusion (84.3%, 274/325) than physicians themselves reported (68.7%, 241/351, *p* < 0.001).

Most surrogates (76.3%, 167/219) emphasized the need for quantitative survival estimates to support discussions, whereas only 23.4% (82/351) of physicians reported using statistical framing (e.g., return of spontaneous circulation rates in in-hospital cardiac arrest) with families (*p* < 0.001). Nearly half of surrogates (47.6%, 131/275) hoped physicians would use decision-support tools, compared to 24.5% (86/351) of physicians who reported doing so (*p* < 0.001) (Table 2).

#### 3.2.3. Decisional Capacity

Physicians perceived that patients had full or partial decisional capacity in 43.9% (154/351) of cases, whereas surrogates reported higher confidence (57.5%, 158/275, *p* < 0.001). Most physicians (94.9%, 333/351) directed consent discussions to surrogates, consistent with the expectations of 64.3% (209/325) of surrogates; however, only 1.4% (5/351) of physicians and 3.4% (11/325) of surrogates supported direct patient involvement (*p* < 0.01). Mentions of advance directives were modestly higher among surrogates (39.6%, 109/275) than physicians (33.9%, 119/351), though this difference was not statistically significant (*p* = 0.139) (Table 2).

#### 3.2.4. Factors Influencing LST Decisions

Among physicians, disease prognosis (96.0%, 337/351) and comorbidities (91.7%, 332/351) were the most commonly cited considerations, followed by patient age (85.2%, 229/351) and patient values (83.5%, 293/351). In contrast, surrogates prioritized patient age (72.0%, 213/296), comorbidities and functional status (41.9%, 124/296), disease prognosis (39.2%, 116/296), and patient values (26.7%, 79/296). Cost considerations were reported by 48.7% (171/351) of physicians but only 4.7% (14/296) of surrogates (*p* < 0.001) (Table 2).

## 4. Discussion

This study compared perceptions and attitudes regarding LST decision-making between physicians and patient surrogates in China. Significant differences were identified in the triggers for LST discussions, decision-making models, preferences for medical fact disclosure, perceptions of patient decisional capacity, and contextual factors influencing LST decisions. While both physicians and surrogates perceived patients as having considerable decisional capacity, they nevertheless preferred to direct consent discussions to surrogates rather than to patients themselves. A notable disconnect was observed in perceived understanding and communication effectiveness. Physicians were more proactive in initiating discussions and emphasized clinical urgency and prognosis. In contrast, surrogates expressed a preference for decision-support tools, more quantitative prognostic data, clearer explanations, and prioritized prognostic factors such as patient age, functional status, and quality of life. These findings reveal a substantial physician–surrogate perception gap in LST decision-making in China, underscoring the need for culturally adapted communication strategies and ethical frameworks. Specifically, there is a need to develop structured decision-support tools that facilitate transparent information exchange and reconcile divergent priorities. The results also highlight the importance of training healthcare providers in culturally sensitive communication that respects familistic norms while promoting patient-centred care and autonomy.

### 4.1. Participant Demographic Characteristics

The physicians’ median experience level (10 years) and the high proportion practicing in tertiary hospitals (89.7%) suggest a cohort well-versed in complex critical care decision-making. Inclusion of physicians across multiple specialties further enriches the diversity of perspectives. These contextual factors are essential in interpreting their reported proactivity in initiating LST discussions and their emphasis on clinical considerations such as prognosis and comorbidities. The literature offers limited insight into the extent to which a surrogate’s knowledge and understanding contribute to the quality of decision-making. In this study, surrogates, mostly middle-aged adults, likely represented children making decisions for critically ill parents. Familiarity with the patient’s values and preferences is likely to be a critical determinant of surrogate decision quality [13]. Prior studies have shown that patients typically choose surrogates based on the closeness of their relationship and the surrogate’s familiarity with their values, preferences, past behaviours, prior decisions, and, importantly, prior experience in making healthcare decisions on their behalf [14].

### 4.2. Comparison of Perceptions and Attitudes Between Surrogates and Physicians

#### 4.2.1. Triggers for Decision-Making and Decision-Making Patterns

Shared decision-making was the most commonly preferred approach among both physicians and surrogates. The strong preference for collaboration among surrogates may also be understood through the lens of their extensive involvement in daily patient care in China. This shared responsibility for the physical work of care likely fosters a greater need for mutual understanding and joint decision-making, distinguishing their experience from that of surrogates in systems where family involvement is more advisory. However, surrogate-led decisions were frequently reported in practice, suggesting that family members often assume primary responsibility for decision-making, even when mutual participation is nominally endorsed. In a previous study conducted in China, 84% of patients expressed a preference to participate in SDM [15], whereas another study reported that 57.02% of Chinese patients engaged in physician-directed decision-making and only 37.79% used SDM [16]. These inconsistencies may be attributable to differences in age, education level, disease trajectory, and knowledge of medications. Approximately 75–90% of surrogates prefer to share responsibility for decision-making with clinicians or to decide after considering clinical recommendations [6,17]. A higher degree of prognostic discordance between clinicians and families regarding hospital survival has been independently associated with greater use of SDM [1]. Factors independently linked to a preference for surrogate-led decisions include lower trust in the ICU physician, male sex, and non-Catholic religious affiliation [6]. The American College of Critical Care Medicine and the American Thoracic Society endorse a broad spectrum of ethically acceptable decision-making approaches, including patient-/surrogate-directed and physician-directed models [17]. Physicians are therefore encouraged to assess each surrogate’s preferred role in decision-making and adapt their level of authority accordingly, taking into account both individual preferences and the clinical context [6].

The timing of diagnostic and prognostic disclosure is critical and remains a major perceived challenge [18]. In our study, physicians were significantly more likely than surrogates to proactively initiate discussions about LST in the context of potential clinical deterioration, often emphasizing prognostic uncertainty. Similar findings were reported in Danish hospitals, where physicians highlighted the importance of clarifying patient preferences for levels of treatment before the onset of severe illness [18]. Society of Critical Care Medicine Guidelines suggested that families benefit from timely and structured communication approaches, with the first family meeting generally recommended within 72 h of admission [19]. Early conversations regarding patient preferences, prior to medical crises, may reduce decisional pressure and foster more open dialogue. Supporting this, Akinori Sasaki and colleagues reported that code status discussions upon admission were associated with reductions in invasive procedures and cardiopulmonary resuscitation (CPR) [20]. Many patients were entirely reliant on clinicians to initiate conversations about prognosis and LST decisions [18]. Training in communication skills is therefore essential [17]. A qualitative study from Canada found that implementation of a serious illness care program prompted behavioural change among hospital clinicians and facilitated more meaningful patient interactions [21]. Similarly, communication workshops for residents were shown to improve specific behavioural competencies during patient encounters [22].

#### 4.2.2. Medical Fact Disclosure

To reach decisions appropriate to the situation, all stakeholders must share a common understanding of the patient’s condition and prognosis [3]. The “reasonable patient standard” refers to the disclosure of information that a reasonable patient would need to make an informed decision, including not only common risks and benefits but also rare, potentially devastating events [5]. For example, at least 74% of patients undergoing prone spinal surgery preferred disclosure of risks associated with low-incidence but life-altering morbidity [23]. In recent years, physicians adopting a participatory style of shared decision-making have been encouraged to move toward a “subjective standard,” in which information is tailored to the specific needs of the individual patient [5]. In our study, the content of communication was largely family-driven and tailored to family requests. Hammami and colleagues reported that extensive information disclosure is often perceived as the norm, and more highly educated patients may be particularly dissatisfied with current disclosure practices [24]. To meet the subjective standard, the scope and focus of informed consent discussions may therefore need to be adjusted [24].

The mismatch between information needs and factual disclosure may explain the knowledge and expectation gaps observed between physicians and surrogates in our study. Deep and colleagues found that 29% of physicians were concerned that decision-makers had a poor understanding of the decisions being made and often disagreed with them [19]. Similarly, Moran et al. reported statistically significant differences between surgeons’ and patients’ expectations regarding outcomes of total knee or hip replacement at six months [25]. Gehlbach and colleagues, surveying patients in the medical ICU and their surrogates, found that respondents’ average predicted survival following in-hospital cardiac arrest with CPR was 71.8%; the higher the predicted survival, the more frequently full code status was preferred [26].

A persistent challenge in this context is the lack of critical information sharing needed to weigh the benefits and risks of treatment. In our study, most surrogates emphasized the importance of quantitative data and expressed a preference for the use of decision-support tools, underscoring their need for structured assistance in complex discussions. Physicians, however, often avoided statistical framing, and some presented information about resuscitation in a scripted, depersonalized, and procedure-focused manner [19]. Mounting evidence suggests that decision aids, such as brochures, web applications, and videos, can improve the quality of decision-making [27]. The critical importance of designing tools that formally incorporate the family role, positioning them as a partner rather than a barrier. The essential need for training clinicians not just on the tool itself, but on the underlying communication principles. Addressing disparities in communication practices may require standardized protocols that integrate additional diagnostic tests, consultations with experts, ethics committee involvement, and other resources to bridge gaps between clinical expertise and surrogate advocacy [3].

#### 4.2.3. Decisional Capacity

On a practical level, seeking informed consent can be challenging, particularly when individuals lose the ability to understand relevant medical information. In our study, physicians generally perceived patients as having partial or limited decisional capacity, whereas surrogates expressed greater confidence in patients’ abilities. This discrepancy reflects differing views on decisional capacity in critical illness and high-stakes scenarios. In a previous study, approximately one in four hospitalized patients lacked decision-making capacity [28]. Among nursing home residents, 82.4% were able to state a simple treatment preference, yet many could not understand treatment alternatives or appreciate the consequences of their choices [29]. During intensive care unit (ICU) stays, 17.8% of critically ill patients lacked decision-making capacity [30]. These findings suggest that a significant proportion of critically ill patients retain at least partial capacity, consistent with the conclusions of our study. Accordingly, healthcare systems are ethically and legally obligated to implement proactive, routine capacity assessments as a standard of care.

Although physicians considered nearly half of their patients to be decision-capable, they overwhelmingly deferred the informed consent process to families in our study, highlighting a gap between the theoretical principle of informed consent and its practical application. Contextual factors further complicate the exercise of patient autonomy [31,32]. Our findings must be interpreted through the lens of specific cultural constructs. In Chinese medical decision-making, family involvement is prioritized, with families often granted the right to be informed and to participate actively in decision-making. This cultural framework places collective interests above individual autonomy [9,33,34]. Filial piety (xiào) obligates children to protect their parents and act in their perceived best interests, which often translates into a duty to shield them from therapeutic disclosure. Protecting patients from psychological distress and fulfilling familial obligations are considered more important than respecting autonomy. Families frequently request that a diagnosis not be disclosed to the patient or that they be informed before the patient [35,36]. This framework helps explain why surrogates in our study frequently assumed primary decision-making responsibility, even when endorsing collaborative models in principle. Their actions can be seen as a moral imperative that can supersede directives for patient autonomy. This fundamental cultural orientation creates a distinct ethical landscape that challenges the direct application of Western-derived SDM models in the Chinese clinical context. Nevertheless, evidence indicates that patients generally prefer to be informed, and that the benefits of disclosure greatly outweigh potential harms [35]. Turnbull and colleagues reported no differences in patients’ willingness to engage in discussions of LST across prognostic scenarios or value statements [37]. Other studies suggest that relatives may choose more aggressive end-of-life interventions than patients would for themselves [38]. Physicians have also reported discomfort when disagreements arise between themselves and patients or families [39]. Authors from Saudi Arabia argue that physicians should respect autonomy by asking patients about their disclosure preferences and clarifying the roles they wish their families to play [31].

In our study, only about one-third of physicians and surrogates were familiar with the concept of advance directives. Unfortunately, as few as 20% of patients who lack decision-making capacity have such directives in place [3]. The presence of advance directives is more likely when surrogates also have them, when surrogates are less religious, and when residents are socially engaged [29]. However, advance care planning (ACP) documentation fails to reflect actual preferences in 70% or more of seriously ill hospitalized older patients [40]. When families cannot determine a patient’s wishes and face the ethical difficulty of judging best interests, they often choose the “safer” path of aggressive care by default [41]. Clinicians, in turn, may risk sustaining life at all costs through disproportionate, unnecessary, or non-beneficial treatments.

#### 4.2.4. Quality of Life and Contextual Features

Maximal supportive care is primarily directed at comfort and dignity, whereas maximal diagnostic and therapeutic care is primarily directed at sustaining life. Most competent patients and families recognize that each goal may be appropriate at different times, and that these two approaches often become mutually exclusive near the end of life [3]. Specific decisions about the focus of care result from a dynamic and complex interplay of multiple factors, including patient characteristics (e.g., comorbidities, age), the preferences and values of surrogates, the perspectives of treating physicians, and even physician demographic factors [42]. In our study, surrogates prioritized patient age, comorbidities, and functional status in daily living, whereas physicians emphasized clinical indicators such as disease prognosis. Barbara JD and colleagues conducted a descriptive, longitudinal ICU study and found that physician expectations for survival and future cognitive status were the only variables consistently and significantly associated with the chosen focus of care [42]. Priorities expressed by surrogates often reflected concerns about functional independence and quality of life following recovery. Similarly, Devnani and colleagues surveyed surrogates of hospitalized patients aged ≥ 65 years and found that 77.8% selected patient well-being as the most important principle, compared with only 21.1% who selected patient preferences [43]. Surrogates were more likely to prioritize patient preferences when advance directives were available [43]. The divergence in priorities between physicians and surrogates underscores the fundamental challenge of shared decision-making. Physicians, trained to prognosticate based on objective clinical data, logically prioritize diagnosis with comorbidities as impact factors. In contrast, surrogates often use age as a holistic proxy for the patient’s potential for recovery, resilience, and their conceptualization of a ‘life worth living.’ Therefore, the most constructive approach is to recognize that the optimal decision-making framework synthesizes both. The ‘best parameter’ is thus a shared understanding that integrates the physician’s evidence-based prognosis with the surrogate’s intimate knowledge of the patient’s values and preferences, thereby aligning medical feasibility with personal meaning. Given the complexity of these decisions, the development of structured guidelines may require leadership not only from physicians but also from other experts, including lawyers, bioethicists, and national guardianship organizations [41].

### 4.3. Limitations

This study has several limitations. First, its exclusive focus on a major metropolitan centre (Beijing) and predominantly tertiary care settings and the surrogate sample was from a single academic medical centre, which limits the generalizability of the findings. The cultural context of family decision-making in China is inherently reflected in these demographics, but practices, resource availability, physician training, and family dynamics may differ considerably in rural areas or lower-tier hospitals. The perspectives captured may not fully represent those of physicians in community or rural hospitals across China’s diverse healthcare landscape, surrogates in different regional contexts where cultural norms and resource availability can vary. The fundamental mismatch in the sampling frames—where physicians were recruited from multiple hospitals across Beijing, while surrogates were drawn from a single EICU—may also be conflated with the contextual differences between a generalized physician population and a highly specific surrogate population. Second, the cross-sectional design captures perceptions at a single point in time and does not allow inferences about causality or the evolution of attitudes during the course of a patient’s critical illness. Our analysis did not control for these potential confounding variables and that future research would benefit from such multivariate modelling. Third, reliance on surveys introduces the possibility of social desirability bias, with participants potentially providing responses they considered socially acceptable rather than their true views. While we identified surrogates as the primary informal decision-makers, we did not collect data on their specific kinship relationship to the patient (e.g., spouse, adult child, parent). It is possible that perspectives on decision-making could vary based on this relationship. Future studies should systematically record this information to explore its potential influence. Although parallel questions were presented to physicians and patient surrogates, conceptual alignment, perfect wording equivalence was not always possible across the two distinct stakeholder groups, and this remains a consideration when interpreting the findings. Fourth, the study did not include the perspectives of patients who may have retained some degree of decision-making capacity, an essential component for a comprehensive understanding of the SDM process. Finally, although the questionnaires were based on prior studies, certain complex constructs, such as “decisional capacity” and “communication quality”, may not have been fully captured by the instruments used.

## 5. Conclusions

This study identifies a significant perception gap between physicians and patient surrogates in multiple domains of shared decision-making for life-sustaining treatment in a Chinese critical care setting. Key discrepancies emerged in decision-making patterns, preferences for information disclosure, assessments of patient decisional capacity, and the factors prioritized when evaluating LST. These findings highlight the urgent need for targeted interventions to bridge this divide. Structured communication protocols, decision aids, the promotion of advance directives, and physician training in cross-cultural communication and preference elicitation represent essential strategies. Ultimately, fostering a decision-making process that aligns clinical expertise with patient and family values is critical to ensuring ethically sound, patient-centred care in China.

## Figures and Tables

**Table 1:** Demographic characteristics of the included physicians.

Characteristics (N =351)	N of Physicians (%)
Age, median years (IQR)	35 (30, 40)
Gender, female	232 (66.1)
Education background, doctor’s degree in medicine	278 (79.2)
Years of medical practice, median years (IQR)	10 (6, 16)
Title	
Senior	90 (25.6)
Median	145 (41.3)
Junior	116 (33.0)
Hospital level, Tertiary hospital	315 (89.7)
Specialty	
Emergency medicine	115 (32.8)
Internal medicine	114 (32.5)
Cardiology	29 (8.3)
Surgery	28 (8.0)
Intensive medicine	28 (8.0)
Work time per week	
40~50 h	153 (43.6)
50~60 h	130 (37.0)
>60 h	68 (19.4)
Performing CPR	
Often	147 (41.9)
Sometimes	120 (34.2)
Occasionally	81 (23.1)
Never	3 (0.9)
Performing CPR during past month	
≥5 times	42 (12.0)
2~4 times	73 (20.8)
≤1 time	68 (19.4)
Previous doctor-patient communication training	284 (80.9)

Abbreviation: IQR: interquartile range; CPR: cadiopulmonary resuscitation.

**Table 2:** Comparison perceptions and attitudes between physicians and surrogates.

Item	Physician, n (%) (N = 351)	Surrogate, n (%) (N = 325)	Pearson χ^2^	*p*
Decision-making pattern			5.246	0.155
Shared decision-making	185/351 (52.7)	144/325 (44.3)		
Surrogate-led	126/351 (35.9)	143/325 (44.0)		
Patient-led	25/351 (7.1)	24/325 (7.4)		
Physician-led	15/351 (4.3)	14/325 (4.3)		
Timing of LST conversation when potential risk	309/351 (88.0)	207/275 (75.3)	17.337	<0.001
Communication content			2.455	0.117
Per surrogate requirement	262/351 (74.6)	225/325 (69.2)		
Per physician work habit	89/351 (25.4)	100/325 (30.8)		
Alternative plan provided	329/351 (93.7)	258/325 (79.4)	30.385	<0.001
Surrogates’ understanding of trauma and prognosis of LST			291.369	<0.001
Excellent	12/251 (3.4)	100/325 (30.8)		
Good	71/351 (20.2)	185/325 (56.9)		
Fair	188/351 (53.6)	36/325 (11.1)		
Poor	71/351 (20.2)	3/325 (0.9)		
Extremely poor	9/351 (2.6)	1/325 (0.3)		
When patient/surrogate confused about LST, actively interpretation	241/351 (68.7)	274/325 (84.3)	22.769	<0.001
If talking about ROSC rate, medical evidence provided	82/351 (23.4)	167/219 (76.3)	153.367	<0.001
Use tools	86/351 (24.5)	131/275 (47.6)	36.439	<0.001
Decisional capacity of patients			92.527	<0.001
Mostly	27/351 (7.7)	66/275 (24.0)		
Partly	127/351 (36.2)	92/275 (33.5)		
Occasionally	197/351 (56.1)	84/275 (30.5)		
Rarely	0 (0.0)	33/275 (12.0)		
Physician obtain inform consent from			101.498	<0.001
Surrogate	333/351 (94.9)	209/325 (64.3)		
Patient	5/351 (1.4)	11/325 (3.4)		
Either one	13/351 (3.7)	105/325 (32.3)		
Mention Advanced Directives	119/351 (33.9)	109/275 (39.6)	2.189	0.139
Factors to be considered on LST				
Patient age	299/351 (85.2)	213/296 (72.0)	17.011	<0.001
Patient comorbidities and daily living status	332/351 (91.7)	124/296 (41.9)	186.309	<0.001
Patient value	293/351 (83.5)	79/296 (26.7)	211.894	<0.001
Disease diagnosis and prognosis	337/351 (96.0)	116/296 (39.2)	246.967	<0.001
Treatment expense	171/351 (48.7)	14/296 (4.7)	152.182	<0.001

Abbreviation: LST: life-sustaining treatment; ROSC: return of spontaneous circulation.

## Data Availability

The data collected for this study contain sensitive personal information of patients, which is protected under strict confidentiality agreements and ethical regulations as approved by our Institutional Review Board. Therefore, public sharing of the raw dataset is not permitted. The raw data can be made available to qualified researchers for the purpose of replicating the results, subject to a formal data sharing agreement that complies privacy laws and ethical requirements.

## Abbreviations

SDM, shared decision-making; LST, life-sustaining treatment; EICU, Emergency Intensive Care Unit; ICU, Intensive Care Unit; DCS, Decisional Conflict Scale; IQR, interquartile range; CPR, cardiopulmonary resuscitation; ACP, advance care planning; POLST, Physician Orders for Life-Sustaining Treatment; IRB, Institutional Review Board; SPSS, Statistical Package for the Social Sciences; ED-ICU, Emergency Department–Intensive Care Unit.

## References

[1] Fleming V., Prasad A., Ge C., Crawford S., Meraj S., Hough C.L., Lo B., Carson S.S., Steingrub J., White D.B. (2023). Prevalence and predictors of shared decision-making in goals-of-care clinician-family meetings for critically ill neurologic patients: A multi-center mixed-methods study. Crit. Care.

[2] Thompson B.T., Cox P.N., Antonelli M., Carlet J.M., Cassell J., Hill N.S., Hinds C.J., Pimentel J.M., Reinhart K., Thijs L.G. (2004). Challenges in end-of-life care in the ICU: Statement of the 5th International Consensus Conference in Critical Care: Brussels, Belgium, April 2003: Executive summary. Crit. Care Med..

[3] Lang F., Quill T. (2004). Making decisions with families at the end of life. Am. Fam. Physician.

[4] Hogan T.M., Richmond N.L., Carpenter C.R., Biese K., Hwang U., Shah M.N., Escobedo M., Berman A., Broder J.S., Platts-Mills T.F. (2016). Shared decision making to improve the emergency care of older adults: A research agenda. Acad. Emerg. Med..

[5] Jonsen A.R., Siegler M., Winslade W.J. (2022). Clinical Ethics, a Practical Approach to Ethical Decisions in Clinical Medicine.

[6] Johnson S.K., Bautista C.A., Hong S.Y., Weissfeld L., White D.B. (2011). An empirical study of surrogates’ preferred level of control over value-laden life support decisions in intensive care units. Am. J. Respir. Crit. Care Med..

[7] Pickard A.S., Knight S.J. (2005). Proxy evaluation of health-related quality of life: A conceptual framework for understanding multiple proxy perspectives. Med. Care.

[8] Gilbar R., Miola J. (2015). One size fits all? On patient autonomy, medical decision-making, and the impact of culture. Med. Law Rev..

[9] Zhang H., Zhang H., Zhang Z., Wang Y. (2021). Patient privacy and autonomy: A comparative analysis of cases of ethical dilemmas in China and the United States. BMC Med. Ethics.

[10] Li S., Xie J., Chen Z., Yan J., Zhao Y., Cong Y., Zhao B., Zhang H., Ge H., Ma Q. (2023). Key elements and checklist of shared decision-making conversation on life-sustaining treatment in emergency: A multispecialty study from China. World J. Emerg. Med..

[11] Ge H.L.S., Li S., Ma Q. (2024). Validation of a checklist to facilitate serious illness conversations in adult emergency in China: A single-centre pilot study. BMC Emerg. Med..

[12] Liang Y., Zhang H., Li S., Ma Q., Ma Q. (2025). Shared decision-making on life-sustaining treatment: A survey of current barriers in practice among clinicians across China. Healthcare.

[13] Shepherd V. (2022). (Re)Conceptualising ‘good’ proxy decision-making for research: The implications for proxy consent decision quality. BMC Med. Ethics.

[14] Dunn L.B., Fisher S.R., Hantke M., Appelbaum P.S., Dohan D., Young J.P., Roberts L.W. (2013). “Thinking about it for somebody else”: Alzheimer’s disease research and proxy decision makers’ translation of ethical principles into practice. Am. J. Geriatr. Psychiatry.

[15] Xu D., Zhang H., Chen Y. (2021). Patients’ views of shared decision making in inflammatory bowel disease: A survey in China. BMC Med. Inf. Decis. Mak..

[16] Hu Q., Feng Z., Zong Q., Wang J., Zheng Z., Feng D. (2023). Analysis of factors that promote the participation of patients with chronic diseases in shared decision making on medication: A cross-sectional survey in Hubei Province, China. BMC Public Health.

[17] Kon A.A., Davidson J.E., Morrison W., Danis M., White D.B., American College of Critical Care Medicine (2016). Shared decision making in ICUs: An American College of Critical Care Medicine and American Thoracic Society policy statement. Crit. Care Med..

[18] Tuesen L.D., Ågård A.S., Bülow H.-H., Fromme E.K., Jensen H.I. (2022). Decision-making conversations for life-sustaining treatment with seriously ill patients using a Danish version of the US POLST: A qualitative study of patient and physician experiences. Scand. J. Prim. Health Care.

[19] Hwang D.Y., Oczkowski S.J.W., Lewis K., Birriel B., Downar J., Farrier C.E., Fiest K.M., Gerritsen R.T., Hart J., Hartog C.S. (2025). Society of Critical Care Medicine Guidelines on Family-Centered Care for Adult ICUs: 2024. Crit. Care Med..

[20] Sasaki A., Hiraoka E., Homma Y., Takahashi O., Norisue Y., Kawai K., Fujitani S. (2017). Association of code status discussion with invasive procedures among advanced-stage cancer and noncancer patients. Int. J. Gen. Med..

[21] Lagrotteria A., Swinton M., Simon J., King S., Boryski G., Ma I.W.Y., Dunne F., Singh J., Bernacki R.E., You J.J. (2021). Clinicians’ perspectives after implementation of the serious illness care program: A qualitative study. JAMA Netw. Open.

[22] Salgaonkar S.V., Kulkarni A.D., Chapane S.P. (2021). Assessment of communication skill during process of preoperative visit and informed consent by anesthesiology residents. J. Anaesthesiol. Clin. Pharmacol..

[23] Corda D.M., Dexter F., Pasternak J.J., Trentman T.L., Nottmeier E.W., Brull S.J. (2011). Patients’ perspective on full disclosure and informed consent regarding postoperative visual loss associated with spinal surgery in the prone position. Mayo Clin. Proc..

[24] Hammami M.M., Al-Jawarneh Y., Hammami M.B., Al Qadire M. (2014). Information disclosure in clinical informed consent: “reasonable” patient’s perception of norm in high-context communication culture. BMC Med. Ethics.

[25] Moran M., Khan A., Sochart D.H., Andrew G. (2003). Expect the best, prepare for the worst: Surgeon and patient expectation of the outcome of primary total hip and knee replacement. Ann. R. Coll. Surg. Engl..

[26] Gehlbach T.G., Shinkunas L.A., Forman-Hoffman V.L., Thomas K.W., Schmidt G.A., Kaldjian L.C. (2011). Code status orders and goals of care in the medical ICU. Chest.

[27] Cox C.E., White D.B., Abernethy A.P. (2014). A universal decision support system. Addressing the decision-making needs of patients, families, and clinicians in the setting of critical illness. Am. J. Respir. Crit. Care Med..

[28] Appelbaum P.S. (2007). Clinical practice. Assessment of patients’ competence to consent to treatment. N. Engl. J. Med..

[29] Allen R.S., DeLaine S.R., Chaplin W.F., Marson D.C., Bourgeois M.S., Dijkstra K., Burgio L.D. (2003). Advance care planning in nursing homes: Correlates of capacity and possession of advance directives. Gerontologist.

[30] White D.B., Curtis J.R., Lo B., Luce J.M. (2006). Decisions to limit life-sustaining treatment for critically ill patients who lack both decision-making capacity and surrogate decision-makers. Crit. Care Med..

[31] Alfahmi M.Z. (2022). Patients’ preference approach to overcome the moral implications of family-centred decisions in Saudi medical settings. BMC Med. Ethics.

[32] Ho M.-J., Alkhal A., Tekian A., Shih J., Shaw K., Wang C.-H., Alyafei K., Konopasek L. (2016). Contextualizing the physician charter on professionalism in Qatar: From patient autonomy to family autonomy. J. Grad. Med. Educ..

[33] Crane M.K., Wittink M., Doukas D.J. (2005). Respecting end-of-life treatment preferences. Am. Fam. Physician.

[34] Meng M., Li X., Zhao J., Hao Y. (2024). When western concept meets eastern culture: Exploring the impact of Confucianism on shared decision-making in China. Asia Pac. J. Oncol. Nurs..

[35] Murgic L., Hébert P.C., Sovic S., Pavlekovic G. (2015). Paternalism and autonomy: Views of patients and providers in a transitional (post-communist) country. BMC Med. Ethics.

[36] Morita T., Oyama Y., Cheng S.-Y., Suh S.-Y., Koh S.J., Kim H.S., Chiu T.Y., Hwang S.J., Shirado A., Tsuneto S. (2015). Palliative care physicians’ attitudes toward patient autonomy and a good death in East Asian countries. J. Pain Symptom Manag..

[37] Turnbull A.E., Krall J.R., Ruhl A.P., Curtis J.R., Halpern S.D., Lau B.M., Needham D.M. (2014). A scenario-based, randomized trial of patient values and functional prognosis on intensivist intent to discuss withdrawing life support. Crit. Care Med..

[38] Sedini C., Biotto M., Crespi Bel’skij L.M., Moroni Grandini R.E., Cesari M. (2022). Advance care planning and advance directives: An overview of the main critical issues. Aging Clin. Exp. Res..

[39] van Bruchem-Visser R.L., de Beaufort I.D., Mattace-Raso F.U.S., Kuipers E.J. (2020). What to do when patients and physicians disagree? Qualitative research among physicians with different working experiences. Eur. Geriatr. Med..

[40] Hickman S.E., Torke A.M., Heim Smith N., Myers A.L., Sudore R.L., Hammes B.J., Sachs G.A. (2021). Reasons for discordance and concordance between POLST orders and current treatment preferences. J. Am. Geriatr. Soc..

[41] Cohen A.B., Wright M.S., Cooney L., Fried T. (2015). Guardianship and end-of-life decision making. JAMA Intern. Med..

[42] Daly B.J., Douglas S.L., O’Toole E., Rowbottom J., Hoffer A., Lipson A.R., Burant C. (2018). Complexity analysis of decision-making in the critically ill. J. Intensive Care Med..

[43] Devnani R., Slaven J.E., Bosslet G.T., Montz K., Inger L., Burke E.S., Torke A.M. (2017). How surrogates decide: A secondary data analysis of decision-making principles used by the surrogates of hospitalized older adults. J. Gen. Intern. Med..

